# A Survey of Spontaneous Antibiotic-Resistant Mutants of the Halophilic, Thermophilic Bacterium *Rhodothermus marinus*

**DOI:** 10.3390/antibiotics10111384

**Published:** 2021-11-11

**Authors:** Sophia Silvia, Samantha A. Donahue, Erin E. Killeavy, Gerwald Jogl, Steven T. Gregory

**Affiliations:** 1 Department of Cell and Molecular Biology, The University of Rhode Island, Kingston, RI 02881, USA; sophie_silvia@my.uri.edu (S.S.); sadonahue@uri.edu (S.A.D.); erin_killeavy@uri.edu (E.E.K.); 2 Department of Molecular Biology, Cell Biology and Biochemistry, Brown University, Providence, RI 02912, USA; gerwald_jogl@brown.edu

**Keywords:** *Rhodothermus marinus*, antibiotic-resistance mutation, ribosome, RNA polymerase, halophile, thermophile

## Abstract

*Rhodothermus marinus* is a halophilic extreme thermophile, with potential as a model organism for studies of the structural basis of antibiotic resistance. In order to facilitate genetic studies of this organism, we have surveyed the antibiotic sensitivity spectrum of *R. marinus* and identified spontaneous antibiotic-resistant mutants. *R. marinus* is naturally insensitive to aminoglycosides, aminocylitols and tuberactinomycins that target the 30S ribosomal subunit, but is sensitive to all 50S ribosomal subunit-targeting antibiotics examined, including macrolides, lincosamides, streptogramin B, chloramphenicol, and thiostrepton. It is also sensitive to kirromycin and fusidic acid, which target protein synthesis factors. It is sensitive to rifampicin (RNA polymerase inhibitor) and to the fluoroquinolones ofloxacin and ciprofloxacin (DNA gyrase inhibitors), but insensitive to nalidixic acid. Drug-resistant mutants were identified using rifampicin, thiostrepton, erythromycin, spiramycin, tylosin, lincomycin, and chloramphenicol. The majority of these were found to have mutations that are similar or identical to those previously found in other species, while several novel mutations were identified. This study provides potential selectable markers for genetic manipulations and demonstrates the feasibility of using *R. marinus* as a model system for studies of ribosome and RNA polymerase structure, function, and evolution.

## 1. Introduction

Extremophilic organisms are important model systems for investigating macromolecular structure, function and evolution. Macromolecular complexes such as the ribosome are important antibiotic targets and their structural studies have significantly advanced our understanding of antibiotic modes of action and mechanisms of antibiotic resistance [1,2,3]. Ribosomes from thermophiles have historically been attractive targets for structural studies due to their greater conformational homogeneity. Such investigations can potentially reveal the basis for adaptation to extreme environments, especially when coupled to genetic approaches. While the most thoroughly examined thermophilic organism is the bacterium *Thermus thermophilus*, studies of other, phylogenetically distant thermophiles could potentially facilitate a comparative approach. This is especially relevant to the extent that species-specific idiosyncrasies, such as differences in DNA repair patterns or codon usage bias, can influence the spectrum of mutants arising. Such idiosyncrasies provide a compelling motivation to explore novel model systems.

*Rhodothermus marinus* R-10^T^ is a Gram-negative, non-motile, non-spore-forming, thermophilic and halophilic bacterium isolated from a submarine hot spring off the coast of Iceland [4]. It grows optimally at a temperature of 65 °C and a salinity of approximately 2%, making it both a model thermophile and a model halophile [5]. Based on 16S rRNA sequence comparisons, the genus *Rhodothermus* has been classified as a member of the *Rhodothermaceae*, branching deeply within the phylum Bacteroidetes, with its closest relative being the mesophilic, extremely halophilic genus *Salinibacter* [6,7]. Other genera of the *Rhodothermaceae* include *Salisaeta* [8], *Rubricoccus* [9], *Rubrivirga* [10], *Longimonas* [11], and *Longibacter* [12]. All members are halophilic and either aerobic or facultatively anaerobic; all but *Rhodothermus* are mesophilic, suggesting that adaptation of *Rhodothermus* to growth at high temperature is a derived rather than a primitive character. Its affinity with *Salinibacter* suggests that adaptation to hypersaline environments predates development of thermostability. This stands in contrast to members of the genus *Thermus*, which form part of a phylum that branches deeply in the universal phylogenetic tree, and for whom thermal adaptation is likely a primitive character. Interestingly, *R. marinus* was isolated from the same environmental sample as the halotolerant IB-21 strain of *Thermus thermophilus* [13], providing an opportunity to compare independently arising adaptations to the same thermal environment. This species is thus of great intrinsic interest from the standpoint of microbial evolution.

We have begun to develop *R. marinus* as a model system for genetic and structural studies of the ribosome and potentially other macromolecular complexes. Like other extremophiles, *R. marinus* has become an important subject of protein structural studies. Notable examples include a novel respiratory complex III [14] and the ribosomal protein uL16 arginyl hydroxylase [15]. Although structures of DNA gyrase, RNA polymerase, and the ribosome from *R. marinus* have yet to be solved, these would seem promising subjects for structural studies given their important roles as targets for major antibiotic classes. Here, we describe antibiotic-resistant mutants of *R. marinus* with alterations in cellular components responsible for gene expression.

*R. marinus* has a number of advantages as a potential model organism for the study of the protein synthesis. In contrast to most other bacteria, the *R. marinus* genome has a single *rrn* operon [16,17], facilitating the isolation of rRNA mutants with pure populations of mutant ribosomes. Early attempts to isolate *E. coli* rRNA mutants were hampered by the presence of seven *rrn* operons such that even dominant mutations arising in a single operon fail to express a selectable phenotype; isolation of such mutants required either expression of rRNA from multi-copy plasmids [18] or deletion of multiple *rrn* operons [19]. In general, ribosome structural studies can be impaired by the complication of mixed populations of mutant and wild-type ribosomes from species with multiple *rrn* operons. More recently, isolation of pure rRNA mutants of *Mycobacterium* spp. [20] or *T. thermophilus* [21] has been facilitated by deletion of one of only two rRNA operons. In the latter organism, isolation of antibiotic-resistant mutants could also arise by efficient homologous recombination between rRNA gene copies during antibiotic selection [22]. While *R. marinus* is not naturally competent for transformation, a method of DNA transfer by electroporation has been described [23] and targeted gene disruptions have been constructed [24]. There is thus significant potential for developing this species as a genetic system [25].

Here, we describe a collection of *R. marinus* mutants having base substitutions in rRNA, or amino acid substitutions or deletions in ribosomal proteins or RNA polymerase. In most instances, these mutations are similar or identical to those found in *T. thermophilus* or mesophilic bacteria. Some mutations, specifically those affecting ribosomal protein uL4, have not been previously observed.

## 2. Results

### 2.1. Spectrum of Antibiotic Sensitivity

Before selecting resistant mutants, we established the range of antibiotics inhibitory to *R. marinus*. Although the sensitivity of *R. marinus* to several antibiotic classes was reported in the initial description of the genus [4], we undertook a more expansive survey. This was done using a simple zone of inhibition disc assay (see Materials and Methods). Results from these assays are indicated in Table 1 and Table S2. We examined antibiotics targeting the ribosome and associated factors, as well as drugs targeting RNA polymerase or DNA gyrase. Assays showing no zone of inhibition were interpreted as indicating resistance.

We found *R. marinus* to be insensitive to all 30S ribosomal subunit antibiotics we tested. We confirmed the previous report of intrinsic resistance of *R. marinus* to the aminoglycosides streptomycin, kanamycin and gentamicin [4] and also found resistance to a number of other aminoglycosides (apramycin, tobramycin, neomycin, paromomycin, neamine, ribostamycin, and hygromycin B). *R. marinus* is also insensitive to the aminocylitols spectinomycin and kasugamycin. We observed resistance to capreomycin, a member of the tuberactinomycins, which binds at the 30S–50S subunit interface, consistent with the previously observed cross-resistance of aminoglycoside-resistant mutants to capreomycin [22,26], suggesting a common basis for insensitivity to both classes of drugs. All other antibiotics showed significant zones of inhibition. We confirmed sensitivity to lincomycin, erythromycin, chloramphenicol, and pristinamycin, all of which bind in or near the peptidyltransferase active site of the 50S subunit. Additional macrolides producing inhibition included the 14-atom macrolides oleandomycin, roxithromycin, and clarithromycin, the 15-atom macrolide azithromycin, and the 16-atom macrolides spiramycin, chalcomycin, tylosin, and carbomycin. The pleuromutilin tiamulin, another inhibitor of peptide bond formation, also inhibits growth of *R. marinus*. In summary, *R. marinus* is resistant to all 30S inhibitors tested and sensitive to all 50S subunit inhibitors tested. Sensitivity was also found to kirromycin and fusidic acid, which target protein synthesis factors EF-Tu and EF-G, respectively. Among non-ribosomal drugs, we found *R. marinus* to be sensitive to the RNA polymerase inhibitor rifampicin and to the DNA gyrase inhibitors ofloxacin and ciprofloxacin, but, as previously reported [4], insensitive to nalidixic acid.

### 2.2. Selection of Spontaneous Mutants

Selection of resistant mutants was attempted with a number of drugs, including the RNA polymerase inhibitor rifampicin, the protein synthesis inhibitors chloramphenicol, lincomycin, erythromycin, spiramycin, tylosin, oleandomycin, thiostrepton, and fusidic acid, and the gyrase inhibitors ofloxacin and ciprofloxacin. Resistant mutants arose on rifampicin, chloramphenicol, lincomyin, erythromycin, spiramycin, tylosin and thiostrepton. No mutants appeared on oleandomycin, fusidic acid, ofloxacin or ciprofloxacin, although a more exhaustive search on a wider range of drug concentrations could potentially reveal mutants resistant to these drugs. Individual isolates were purified and analyzed by sequencing the genes known from previous studies to be the likely sites of mutations. Based on our own studies with *T. thermophilus* [22], we had no reason to expect major differences in the general location of these mutations compared with mesophilic species.

### 2.3. Rifampicin-Resistance (Rif^R^) Mutations in the RNA Polymerase β Subunit

**Figure 1:**
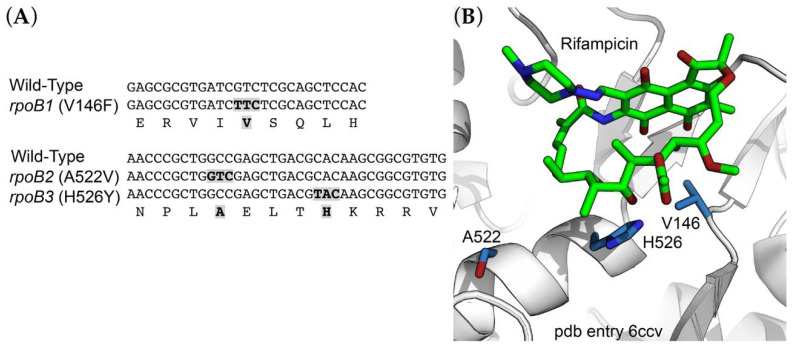
Sites of Rif^R^ resistance mutations. (**A**) Alignment of a segment of RNA polymerase β-subunit sequence encompassing sites of Rif^R^ mutations. (**B**) Structure of the rifampicin binding site within *Mycobacterium smegmatis* RNA polymerase [28], showing positions corresponding to sites of Rif^R^ mutations in the *R. marinus* RNA polymerase β subunit.

Rifampicin inhibits bacterial transcription by binding to the β subunit of RNA polymerase, and mutations conferring resistance are generally found in *rpoB*, the gene encoding the β subunit. We identified three independent *R. marinus* Rif^R^ alleles of *rpoB* (locus tag RMAR_RS05525). These included *rpoB1* (GTC to TTC) producing the amino acid substitution V146F; *rpoB2* (GCC to GTC) producing A522V; and *rpoB3* (GCA to TCA) producing H526Y (Figure 1A; *E. coli* amino acid residue numbering). Mutations at these positions have been found in other species (reviewed by [27]) and are located in or near the rifampicin binding site (Figure 1B; pdb entry 6ccv [28]).

### 2.4. Thiostrepton-Resistance Mutations in the 50S Ribosomal Subunit

**Figure 2:**
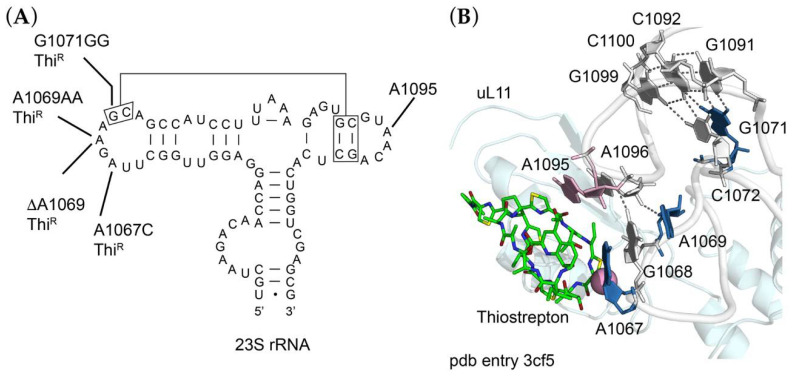
Sites of mutations in 23S rRNA (**A**) Secondary structure of *Rhodothermus marinus* 23S rRNA in the vicinity of the thiostrepton binding site, indicating in blue the sites of single base substitution (A1067C), single base deletion (ΔA1069) and single nucleotide insertions (A1069AA and G1071GG), each conferring thiostrepton-resistance (Thi^R^). (**B**) Three-dimensional structure of the thiostrepton binding site in the crystal structure of the *Deinococcus radiodurans* 70S ribosome-thiostrepton complex (pdb entry 3cf5) [29]. Thiostrepton (green sticks); ribosomal protein uL11 (palecyan); 23S rRNA, white cartoon backbone with skyblue residues mutated to confer resistance in *R. marinus*. A1095 is colored pink. The 2′OH of A1067, site of methylation by resistance methyltransferases, is shown as a pink sphere.

Thiostrepton is a peptide antibiotic that acts as an inhibitor of EF-G-dependent translocation and binds to the 50S ribosomal subunit in a cleft formed by ribosomal protein uL11 and its binding site on 23S rRNA. The uL11 binding site itself is formed by two 23S rRNA loops (residues 1065–1073 and 1093–1098) brought together by a series of tertiary interactions (Figure 2A). Thiostrepton makes direct contact with A1067 and A1095 in these two loops and resistance can occur by base substitutions at either position, by enzymatic 2′-*O*-methylation of A1067, or by amino acid substitutions, deletions or insertions in uL11 (reviewed by [29]). We sequenced *rrlA* encoding 23S rRNA (locus tag RMAR_RS000900) and *rplK* encoding uL11 (locus tag RMAR05505) of the Thi^R^ mutants. While we did not identify any changes in uL11, all mutants were found to have alterations at or near A1067 of 23S rRNA (Figure 2). These included an A1067C transversion, a deletion of A1069 (ΔA1069), a duplication of A1069 (A1069AA), or a duplication of G1071 (G1071GG). As seen in the *Deinococcus radiodurans* 50S ribosomal subunit-thiostrepton complex [29], A1067 is within a few Ångstroms of thiostrepton (Figure 2B), such that 2′-*O*-methylation could sterically block drug binding. The mechanism of resistance conferred by a base substitution at this position is not clear, but it presumably creates sufficient local distortion to decrease binding affinity. In contrast, changes at A1069 and G1071 are further away and must act more indirectly; residues 1067, 1068 and 1069 form a continuous base stack such that duplications or deletions of any of these residues could lead to repositioning of A1067. G1071 participates in a base triple with the G1091-C1100 base pair, which in turn is stacked on a base triple involving C1072, C1092 and G1099. Duplication of G1071 could destabilize the tertiary interaction between the two loops, thereby repositioning either A1067 or A1095, or both. This tertiary interaction is more directly stabilized by single hydrogen bonds between the N2 of G1068 and the O3′ of A1095, and between the O4′ of A1069 and the O2′ of A1096. Duplication or deletion of A1069 could easily disrupt these interactions and influence the positioning of A1067 and A1095.

### 2.5. Base Substitutions of 23S rRNA Residues in and around the Ribosomal Peptidyltransferase Active Site

The peptidyltransferase active site is situated deep within the 50S ribosomal subunit, and X-ray crystal structures of the bacterial ribosome in complex with transition state analogs indicate that all direct contacts with substrates in the active site involve residues of 23S rRNA [30]. Chloramphenicol, macrolides, lincosamides, streptogramin B, and various other antibiotics bind at or near this site, and in many cases mutually compete for binding (for an extensive review of antibiotic action, see [31]). While chloramphenicol, lincosamides, streptogramin B and some macrolides are bona fide peptidyltransferase inhibitors, other macrolides impair elongation of the nascent peptide by occluding the peptide exit channel; more recent studies have shown that macrolides inhibit the synthesis of a subset of proteins rather than inhibiting global translation (reviewed by [32]).

Chloramphenicol-resistant (Chl^R^) mutants of *R. marinus* were readily identified (Table 2) and occur at multiple sites within the central loop of 23S rRNA domain V, corresponding to the peptidyltransferase active site. These included A2059G, G2061C, A2062C, A2453C, U2500A, A2503C, A2503G, and U2504G. Each of these mutations is consistent with our previous studies with *T. thermophilus*. The most abundant mutation was A2058G, perhaps one of the most frequently described antibiotic-resistance mutation in the ribosome and was found independently in selections for erythromycin- (Ery^R^), spiramycin- (Spi^R^), tylosin- (Tyl^R^), and lincomycin-resistant (Lin^R^) mutants.

**Figure 3:**
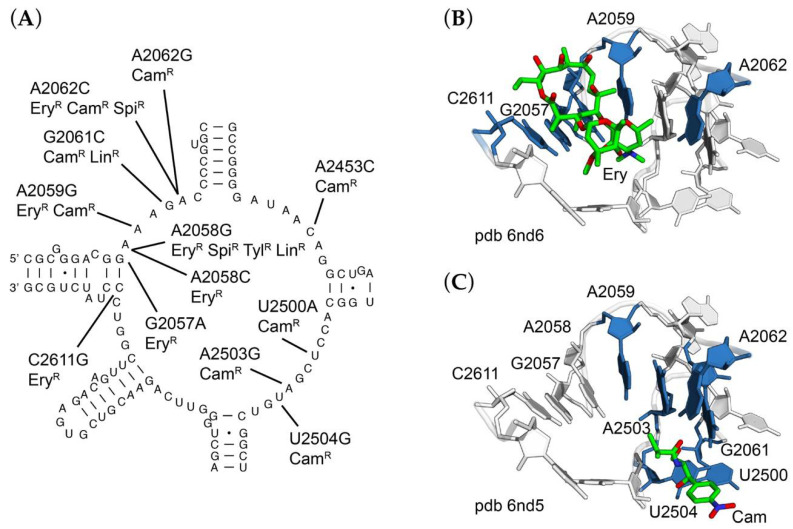
Sites of mutations in or near the peptidyltransferase active site of 23S rRNA. (**A**) Secondary structure of Rhodothermus marinus central loop of domain V of 23S rRNA, with sites of mutations indicated, along with resistance phenotypes conferred. Three-dimensional structure of the peptidyltransferase center of *Thermus thermophilus* 70S ribosome in complex with (**B**) erythromycin (pdb entry 6nd6) and (**C**) chloramphenicol (pdb entry 6nd5). For panels (**B**,**C**), erythromycin or chloramphenicol are shown as green sticks; sites of mutations conferring resistance are colored blue.

In addition, several double mutants were identified. These include A2453C/U2500A, selected on chloramphenicol; G2057A/A2062G, selected on erythromycin; and A2062G/A2503G, selected on spiramycin. The A2062G/A2503G double substitution is especially interesting in that A2062 and A2503 form a symmetrical base pair with one another via their Hoogsteen faces with two N6-N7 hydrogen bonds; either mutation alone could be isolated on chloramphenicol, while A2062G was isolated on either chloramphenicol or erythromycin (Figure 3). The appearance of multiple base substitutions is unusual and not easily explained but could be indicative of a high natural mutation frequency or stress-induced mutagenesis. Our protocols for selection of mutants are designed to minimize exposure to drug. Further studies will be needed to address this question. All of these residues are within close proximity to the corresponding drug binding sites as observed in *T. thermophilus* ribosome crystal structures (Figure 3B,C) [33].

### 2.6. Deletions in a Conserved Loop of Ribosomal Protein uL4 Conferring Erythromycin Resistance

While the peptidyltransferase active site is composed of rRNA, several proteins have globular domains situated on the subunit surface and extended structures that approach the active site, including uL2, uL3, uL4, and bL27 [30]. Proteins uL4 and uL22 form part of the polypeptide exit channel, near the binding site for macrolides. In a number of pathogenic organisms, resistance to erythromycin or other macrolides has been found to result from mutations in *rplD* or *rplV*, encoding 50S ribosomal proteins uL4 and uL22, respectively (reviewed by [34]). A large collection of mutations in both these proteins in *E. coli* reveal a wide range of mutations conferring macrolide resistance [35].

We identified a number of *R. marinus* mutants selected for resistance to several macrolides, including erythromycin, spiramycin, and tylosin, and sequenced both *rplD* and *rplV* of each of these. No mutations in *rplV* were found in any mutant. Several Ery^R^ mutants were found to have deletions within *rplD* (locus tag RMAR_RS04205), initially noted by the diminished size of PCR products as viewed by agarose gel electrophoresis. Sequencing confirmed that the *rplD* genes of these mutants had deletions in the region corresponding to the loop of uL4 that extends toward the peptidyltransferase center and polypeptide exit channel where erythromycin binds.

**Figure 4:**
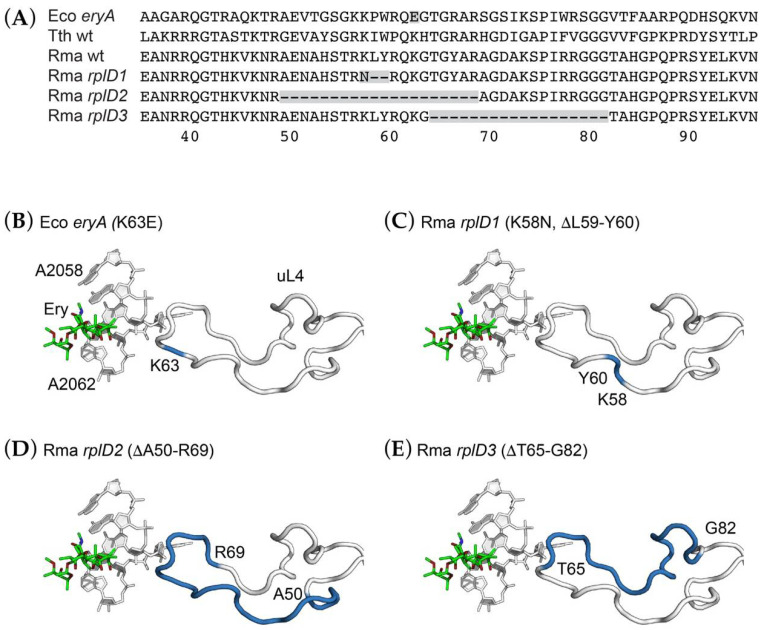
Erythromycin-resistance mutations affecting ribosomal protein uL4. (**A**) Partial sequence alignment of ribosomal proteins uL4 from *Escherichia coli* (Eco), *Thermus thermophilus* (Tth), and *Rhodothermus marinus* (Rma) highlighting sights of erythromycin-resistance mutations. (**B**–**E**) Three-dimensional structure showing the interaction with the extended loop of ribosomal protein uL4 and the erythromycin binding site of 23S rRNA, illustrated using the *Thermus thermophilus* 70S ribosome-erythromycin complex (pdb entry 6nd6). For clarity, only several 23S rRNA nucleotides and the extended loops of uL4 are shown. Residues mutated are colored blue. (**B**) The *eryA* K63E mutation from *E. coli*. (**C**) The *R. marinus rplD1* (K58N, ΔL59-Y60) mutation. (**D**) The *R. marinus rplD2* (ΔA50-R69) mutation. (**E**) The *R. marinus rplD3* (ΔT65-G82) mutation. Each of these mutations is expected to perturb RNA conformation in the macrolide binding site.

Three distinct deletion mutations were identified in *rplD* (Figure 4). One of these, an out-of-frame deletion of the sequence 5′-GCTGTA-3′, results in both a K58N substitution and the deletion of L59 and Y60. A second allele, consisting of a 60-base pair, 20-amino acid deletion extending from A50 to R69, appears to be the result of homologous recombination between two short, direct repeats of the sequence 5′-CGGGCCG-3′. The third allele, a 54-bp, 18-amino acid deletion removing T65 to G82, appears to be the result of homologous recombination between direct repeats of the sequence 5′-GGTACGG-3′ (Figure S1). The original *E. coli* erythromycin-resistance mutation, *eryA* [36], resulted in a K63E substitution in the same region of uL4 [37]. Several site-directed mutagenesis studies of *E. coli* uL4 have demonstrated the malleability of this extended loop and its role in macrolide-ribosome interactions [38,39,40]. However, we are not aware of spontaneous deletions of uL4 of this magnitude having been previously reported.

## 3. Discussion

*R. marinus* is both extremely thermophilic and halophilic, making it quite distinct physiologically from other organism that have been the subject of study of antibiotic action and mechanisms of resistance. Nevertheless, it is remarkable that many of the same mutations that confer antibiotic resistance in mesophiles also confer resistance in extremophiles such as *T. thermophilus* and *R. marinus*. Thus, the majority of the mutations we identified in this study are identical to those observed in a wide range of organisms, though our collection of *R. marinus* mutants is larger than most reported in individual species in a single study. Most of the base substitutions in the peptidyltransferase active site are at positions in close contact with various antibiotics, as observed in crystal structures of ribosome-antibiotic complexes (reviewed by [3]). The same can be said of the RNA polymerase mutants we found. This similarity is no doubt the result of the extreme conservation of rRNA sequence and structure in ribosome functional centers, and the catalytic center of RNA polymerase.

A number of high-resolution structures of the *T. thermophilus* RNA polymerase have been determined in various complexes [41,42,43], including with rifampicin [44]. More recently, the structure of the *M. smegmatis* RNA polymerase-rifampicin complex has been solved [28]. Of the *R. marinus* RNA polymerase residues mutated in Rif^R^ mutants, V146 and H526 are both quite conserved, whereas A522 is less so. Substitutions at H526 are among the more frequently observed Rif^R^ mutations in *Mycobacterium tuberculosis* [27]. As seen in the *M. smegmatis* RNA polymerase-rifampicin complex [28], all three residues are within the rifampicin binding site, although A522 is somewhat removed from rifampicin such that the mechanism of resistance caused by substitutions at this position is unclear (Figure 1B). It is perhaps worth noting that this position is a Ser in *E. coli*, *M. tuberculosis*, and *T. aquaticus*. While none of these residues make direct contact with the drug, their exchange with bulkier residues is likely to have a strong steric effect on drug binding.

Crystal structures of antibiotics bound to the peptidyltransferase center help to explain their mechanism of action [3]. As illustrated in Figure 3, base substitutions conferring drug resistance are components of the drug binding site. In the case of erythromycin-resistance, sites of mutations are located in close proximity to the drug, and the same is true for chloramphenicol-resistance. Consistent with this observation, mutations simultaneously conferring resistance to both drugs are located between the two drug binding sites. Further, crystal structures of ribosomes containing antibiotic-resistance mutations show small perturbations in local structure or indicate a loss of ribosome-drug contact [45]. In contrast to sites of rRNA mutations, ribosomal protein mutations are less similar across species. This is probably in part due to the lower conservation of ribosomal protein sequences, but more likely the lack of conservation at the DNA sequence level, which constrains the specific mutations that can occur. The deletion mutations in *rplD* observed in *R. marinus* are the result of recombination between short, fortuitously repeated DNA sequences. Synonymous codons at either of these repeats would presumably prevent these particular deletions from arising.

The finding of deletion mutations in uL4 is consistent with previously identified mutations in this protein. The original *E. coli eryA* allele of *rplD* was found to result in a single amino acid substitution, K63E [37] (Figure 4B). This mutation was subsequently found to cause an increase in the frequency of translational errors, including misreading errors and frameshifting [46]. While an assay for measuring translational accuracy does not yet exist for *R. marinus*, it should be possible to assess their effects by reconstructing the analogous deletions in *E. coli rplD*. While there is as yet no high-resolution structure of the *R. marinus* ribosome, structures of ribosomes from a variety of organisms indicate that the loop subjected to these deletion mutations is located in close proximity to 23S rRNA residues involved in erythromycin binding. A wide variety of amino acid substitutions as well as small deletions (1 to several residues) in this loop have been found in multiple species.

The lack of direct contact between uL4 and erythromycin demands consideration of an indirect mechanism of resistance, such as a local destabilization of rRNA conformation. The deletions could be mapped onto the *T. thermophilus* ribosome crystal structure and it is clear that they must abolish any direct contact with 23S rRNA in the erythromycin binding site (Figure 4C–E). This notion is consistent with a previous chemical probing study [47] and subsequent cryo-electron microscopic reconstruction [48] of the *E. coli* uL4 mutant, both of which showed significant structural distortion from a single amino acid substitution. We would expect the *R. marinus* deletions to make substantially greater distortions. A crystal structure of an archaeal 50S subunit bearing a 3 amino acid deletion in ribosomal protein uL22 shows the repositioning of several bases in the peptidyltransferase center [45]. Remarkably, the extended loops of both uL4 and uL22 of *E. coli* are dispensable for protein synthesis and growth but make important contributions to the kinetics and fidelity of 50S subunit assembly [40]. We expect that the deletions within uL4 will show extensive distortions, and future cryo-EM reconstructions of the *R. marinus* ribosome could be effective in testing this hypothesis.

The finding that *R. marinus* is resistant to aminoglycosides, capreomycin, kasugamycin, and spectinomycin, was unexpected. One possible explanation is an inability to import these drugs into the cell, and given the close structural relationship of the aminoglycosides, a common uptake mechanism seems plausible. Members of the *Bacteroides* genus are inherently resistant to aminoglycosides due to lack of an oxygen- or nitrate-dependent electron transport system [49]. This explanation is insufficient to explain resistance of *R. marinus*, which is obligately aerobic. Another possibility is natural variation in ribosome structure. Inspection of the 16S rRNA sequence revealed variation in the aminoglycoside binding site, a A1409-U1491 base pair, as opposed to the C1409-G1491 more frequently found in bacterial 16S rRNAs. Data retrieved from the Comparative RNA Web Site and Project database (http://www.rna.ccbb.utexas.edu; accessed on 9 August 2020) [50] indicate that among bacterial sequences, a C-G pair is found in 84.5% of bacterial 16S rRNA sequences, while an A-U base pair is found in 12.6% of sequences. These data also indicate that the adjacent base pair, 1410–1490, is C-G in *R. marinus*, a sequence found in only 0.1% of bacterial 16S rRNA sequences. The mesophilic extreme halophile *Salinibacter ruber*, whose closest relative is *R. marinus*, is also resistant to kanamycin and its 16S rRNA also has the A1409-U1491 base pair [6,7]. Based on secondary structure models retrieved from the RNAcentral database (http://rnacentral.org/; accessed on 9 August 2020) [51], this same A-U base pair is present in all members of the *Rhodothermaceae*. Whether or how either of these base pair identities might influence the aminoglycoside binding site is not obvious. Previous studies have found the aminoglycoside-resistance mutations C1409G of yeast mitochondrial 17S rRNA [52], or a G1491A of *Tetrahymena thermophila* 18S rRNA [53]. This would also explain the resistance to capreomycin, as these mutations confer cross-resistance to tuberactinomycins. In the absence of an in vitro protein synthesis system for *R. marinus*, it is not yet possible to distinguish between these two possible explanations for resistance to aminoglycosides and capreomycin.

## 4. Materials and Methods

### 4.1. Strains and Growth Conditions, Assessment of Antibiotic Sensitivity, and Isolation of Mutants

All mutants were derived from *R. marinus* R-10^T^ ATCC 43812/DSM 4252 [4], which was a kind gift of JHD Cate, University of California, Berkeley. *R. marinus* was cultivated in liquid TEM medium (ATCC Medium 1598) containing 2% NaCl (referred to hereafter as TEMS medium) or on TMG medium containing 2% NaCl (referred to hereafter as TMGS medium). TMG medium consists of TEM lacking phosphate buffer and solidified with gelrite at a concentration of 1.1%. All cultures were grown at 65 °C under aerobic conditions with vigorous aeration at 200 rpm in a New Brunswick Innova 42 Shaker Incubator. Overnight cultures were typically cultivated in 20 mL of medium in 125 mL baffled culture flasks (Corning).

To assay antibiotic sensitivity, 100 μL of a saturated overnight culture grown in TMGS broth was spread-plated onto TMGS plates. A disc infused with 100 μg of antibiotic was placed onto the surface of the plate, which was then incubated at 65 °C overnight; zones of inhibition were subsequently measured. Spontaneous mutants were selected by spreading approximately 10^9^ cells from a saturated overnight culture onto TMGS plates containing various antibiotic concentrations; chloramphenicol, 25, 50, or 100 μg/mL; erythromycin, 50, 100, or 200 μg/mL; tylosin, 100 μg/mL; spiramycin, 100 μg/mL; lincomycin, 100 μg/mL; thiostrepton, 25, 50, 100, or 200 μg/mL; rifampicin, 50, 100, or 200 μg/mL. Mutants were purified by restreaking onto TMGS medium containing antibiotic at the same concentration used in selection, then a second time on antibiotic-free TMGS. Mutants were never exposed to antibiotic after the initial single colony isolation. Single colonies were used to inoculate TEMS medium and shaken at 65 °C to saturation. Mutants were archived as 25% glycerol stocks at −80 °C.

### 4.2. Identification of Mutations

Chromosomal DNA (gDNA) was prepared using Wizard Genomic DNA Kit (Promega, Madison, WI, USA). Oligonucleotide primers were synthesized by IDT and are described in Supplementary Table S1. All PCR reactions were performed using OneTaq DNA polymerase (New England Biolabs, Ipswich, MA, USA). Sanger sequencing of PCR products was performed by the Genomics and Sequencing Center at The University of Rhode Island. The *rrnA* operon encoding 16S rRNA (locus tag RMAR_RS00885), tRNA^Ile^ (locus tag RMAR_RS00890), tRNA^Ala^ (locus tag RMAR_RS00895), 23S rRNA (locus tag RMAR_RS00900), and 5S rRNA (locus tag RMAR_ RS00905), was amplified using primers Rma_rrnA_f3 and Rma_rrnA_r3, with a 58 °C annealing temperature and a 6 min extension time. The *rplD* gene encoding ribosomal protein uL4 (locus tag RMAR_RS04205) was amplified using primers Rma_rplD_f1 and Rma_rplD_r1, with a 52.5 °C annealing temperature and a 1 min extension time. The *rplV* gene encoding ribosomal protein uL22 (locus tag RMAR_RS04225) was amplified using primers Rma_rplV_f1 and Rma_rplV_r1, with a 49 °C annealing temperature and a 1 min extension time. The *rplK* gene encoding ribosomal protein uL11 (locus tag RMAR_RS05505) was amplified using primers Rma_rplK_f1 and Rma_rplK_r1, with a 60 °C annealing temperature and a 1 min extension time. The *rpoB* gene encoding the β-subunit of RNA polymerase (locus tag RMAR_RS05525) was amplified using primers Rma_rpoB_f1 and Rma_rpoB_r1, with a 49 °C annealing temperature and a 4 min extension time. Sequencing of the *rrlA* gene encoding 23S rRNA was performed using primers Rma_rrnA_f4, Rma_rrnA_f7, Rma_rrnA_f8, Rma_rrnA_r5, Rma_rrnA_r6, Rma_rrnA_r7.

## 5. Conclusions

Antibiotic sensitivity spectra and patterns of cross resistance can potentially be informative from both phylogenetic and ribosome structure-function perspectives. Extensive surveys of antibiotic-resistance mutations have been conducted for only a handful of species, making broad generalizations difficult. In this study, we have isolated and characterized a number of antibiotic-resistant mutants of a single species, potentially allowing direct comparisons of mutant phenotypes. Importantly, we find that mutations arising in a thermophilic-halophilic species closely resemble those found in mesophilic species, consistent with the extreme sequence conservation (and by implication, structural conservation) of antibiotic-binding sites in RNA polymerase and the ribosome. Surprising was the inherent resistance of this species to a range of structurally-unrelated 30S subunit inhibitors. The basis for this resistance remains to be determined. The ability to readily isolate rRNA mutations in this species makes it a candidate for future structural studies to address this question.

## Figures and Tables

**Table 1:** Antibiotic sensitivity spectrum of *R. marinus* R-10^T^. Sensitivity was assessed using a disc assay to measure zones of inhibition.

Antibiotic Class, (Target)	Antibiotic	Response
aminoglycoside (ribosome, 30S)	streptomycin, apramycin, hygromycin B, gentamicin, neomycin, neamine, ribostamycin, kanamycin, tobramycin, paromomycin	resistant
aminocyclitols (ribosome, 30S)	kasugamycin, spectinomycin	resistant
tuberactinomycin (ribosome, 70S)	capreomycin	resistant
14-atom macrolides (ribosome, 50S)	erythromycin, oleandomycin, roxithromycin, clarithromycin	sensitive
15-atom macrolide (ribosome, 50S)	azithromycin	sensitive
16-atom macrolides (ribosome, 50S)	spiramycin, chalcomycin, tylosin, carbomycin	sensitive
lincosamides (ribosome, 50S)	lincomycin, clindamycin	sensitive
streptogramin B (ribosome, 50S)	pristinamycin	sensitive
pleuromutilin (ribosome, 50S)	tiamulin	sensitive
amphenicol (ribosome, 50S)	chloramphenicol	sensitive
thiopeptide, (ribosome, 50S)	thiostrepton	sensitive
elfamycin (EF-Tu) ^1^	kirromycin	sensitive
fusidane (EF-G) ^2^	fusidic acid	sensitive
rifamycin (RNA polymerase)	rifampicin	sensitive
quinolone (DNA gyrase)	nalidixic acid	resistant
fluoroquinolones (DNA gyrase)	ofloxacin, ciprofloxacin	sensitive

^1^ EF-Tu, protein synthesis elongation factor Tu. ^2^ EF-G, protein synthesis elongation factor G.

**Table 2:** Mutations identified in this study. Abbreviations: Rep Strain, representative strain; Chl, chloramphenicol; Ery, erythromycin; Spi, spiramycin; Tyl, tylosin; Lnc, lincomycin; Thi, thiostrepton; Rif, rifampicin.

Allele	Rep Strain	Mutation	Selection
*rpoB1*	SOP89	RNA pol β subunit-V146F	Rif50, 100
*rpoB2*	SOP90	RNA pol β subunit-A522V	Rif50, 100
*rpoB3*	SOP91	RNA pol β subunit-H526Y	Rif50
*rrlA1*	SOP9	23S rRNA-G2057A	Ery50, 100, 200
*rrlA2*	SOP23	23S rRNA-A2058C	Ery200
*rrlA3*	SOP11	23S rRNA-A2058G	Ery50, 100, 200/Spi100/Tyl100/Lnc100
*rrlA4*	SOP26	23S rRNA-A2059G	Ery200/Chl100
*rrlA5*	SOP56	23S rRNA-G2061C	Chl100/Lnc100
*rrlA6*	SOP29	23S rRNA-A2062C	Chl25/Ery50/Spi100
*rrlA7*	SOP38	23S rRNA-A2062G	Chl25/Ery50
*rrlA8*	SOP74	23S rRNA-A2453C	Chl25
*rrlA9*	SOP5	23S rRNA-U2500A	Chl50
*rrlA10*	SOP4	23S rRNA-A2503C	Chl50
*rrlA11*	SOP1	23S rRNA-A2503G	Chl25, 50
*rrlA12*	SOP3	23S rRNA-U2504G	Chl25, 50
*rrlA13*	SOP14	23S rRNA-U2611G	Ery50
*rrlA14*	SOP7	23S rRNA-A2453C/U2500A	Chl50
*rrlA15*	SOP24	23S rRNA-G2057A/A2062G	Ery200
*rrlA16*	SOP60	23S rRNA-A2062G/A2503G	Spi100
*rrlA17*	SOP72	23S rRNA-A1067C	Thi200
*rrlA18*	SOP79	23S rRNA-ΔA1069	Thi100
*rrlA19*	SOP73	23S rRNA-A1069AA	Thi200
*rrlA20*	SOP77	23S rRNA-G1071GG	Thi100
*rplD1*	SOP57	uL4-K58N, ΔL59-Y60	Ery100, 200
*rplD2*	SOP25	uL4-ΔA50-R69	Ery200
*rplD3*	SOP16	uL4-ΔT65-G82	Ery100

## Supplementary Materials

The following are available online at https://www.mdpi.com/article/10.3390/antibiotics10111384/s1, Figure S1: Generation of *rplD* erythromycin-resistance mutations [54]; Table S1: Oligonucleotides used in this study [17]; Table S2: Antibiotic zones of inhibition.
