# Supplementary material for: A Survey of Spontaneous Antibiotic-Resistant Mutants of the Halophilic, Thermophilic Bacterium Rhodothermus marinus
Source: Antibiotics (Basel). 2021 Nov 11;10(11):1384. doi: 10.3390/antibiotics10111384 (PMC8614978; doi:10.3390/antibiotics10111384)
Supplement: Supplementary file 1 [file antibiotics-10-01384-s001.zip › antibiotics-1407193-supplementary.pdf]

Supplemental Materials

Figure S1. Generation of *rplD* erythromycin-resistance mutations. Pairwise alignment of wild-type *R. marinus rplD* gene and uL4 protein sequences with the corresponding sequences from each of the three erythromycin-resistant mutants SOP57 (*rplD1*), SOP25 (*rplD2*), and SOP16 (*rplD3*). The sequence deleted and mutated in *rplD1* is highlighted in grey, as are the direct repeat sequences involved in homologous recombination to produce the *rplD2* and *rplD3* alleles. Sequences were aligned using Clustal Omega (https://www.ebi.ac.uk/Tools/msa/clustalo/) [54].

WT

```
AAGAACCGGGCCGAAAACGCCCACAGCACCCGCAAGCTGTACCGCCAGAAAGGTACGGGTTACGCCCGGGCCGGCGATGCCAAGTCGCCCATTCGTCGGGGGGGTGGTACGGCCCACGGG
 K  N  R  A  E  N  A  H  S  T  R  K  L  Y  R  Q  K  G  T  G  Y  A  R  A  G  D  A  K  S  P  I  R  R  G  G  G  T  A  H  G
          50                      58 59 60                            70                            80
```

rplD1 (K58N,ΔA50-R69)

```
AAGAACCGGGCCGAAAACGCCCACAGCACCCGCAACCGCCAGAAAGGTACGGGTTACGCCCGGGCCGGCGATGCCAAGTCGCCCATTCGTCGGGGGGGTGGTACGGCCCACGGG
 K  N  R  A  E  N  A  H  S  T  R  N  R  Q  K  G  T  G  Y  A  R  A  G  D  A  K  S  P  I  R  R  G  G  G  T  A  H  G
          50                      K58N,ΔL59-Y60                 70                            80
```

WT

```
AAGAACCGGGCCGAAAACGCCCACAGCACCCGCAAGCTGTACCGCCAGAAAGGTACGGGTTACGCCCGGGCCGGCGATGCCAAGTCGCCCATTCGTCGGGGGGGTGGTACGGCCCACGGG
 K  N  R  A  E  N  A  H  S  T  R  K  L  Y  R  Q  K  G  T  G  Y  A  R  A  G  D  A  K  S  P  I  R  R  G  G  G  T  A  H  G
          50                            60                            70                            80
```

rplD2 (ΔA50-R69)

```
AAGAACCGGGCCGGCGATGCCAAGTCGCCCATTCGTCGGGGGGGTGGTACGGCCCACGGG
 K  N  R  A  G  D  A  K  S  P  I  R  R  G  G  G  T  A  H  G
          50 71                         80
```

WT

```
AAGAACCGGGCCGAAAACGCCCACAGCACCCGCAAGCTGTACCGCCAGAAAGGTACGGGTTACGCCCGGGCCGGCGATGCCAAGTCGCCCATTCGTCGGGGGGGTGGTACGGCCCACGGG
 K  N  R  A  E  N  A  H  S  T  R  K  L  Y  R  Q  K  G  T  G  Y  A  R  A  G  D  A  K  S  P  I  R  R  G  G  G  T  A  H  G
          50                            60          64                70                            80          84
```

rplD3 (ΔT65-G82)

```
AAGAACCGGGCCGAAAACGCCCACAGCACCCGCAAGCTGTACCGCCAGAAAGGTACGGCCCACGGG
 K  N  R  A  E  N  A  H  S  T  R  K  L  Y  R  Q  K  G  T  A  H  G
          50                            60          64    84
```

Table S1. Oligonucleotides used in this study. Locus tags and genome coordinates are taken from the accession number NC_013501.1 [17].

Name	Sequence (5'-3')	Gene (Gene Product)	Locus Tag	Genome Coordinates
Rma_rrnA_f1	GCGAATCTGGCCGGAACCGAACAGGC	*rrsA* (16S rRNA)	RMAR_RS00885	186847-186872
Rma_rrnA_f2	CATGCGGCTGAAAGGAACCCGGCCG	*rrsA* (16S rRNA)	RMAR_RS00885	186751-186775
Rma_rrnA_f3	CGACGGTTGATGTAAATTGCCGGTCC	*rrsA* (16S rRNA)	RMAR_RS00885	187033-187058
Rma_rrnA_f4	GTAAACCGAGCAAAGCAAGGAG	*rrlA* (23S rRNA)	RMAR_RS00900	189144-189165
Rma_rrnA_f5	TAGCCCTTAATTATAAGGTATCGG	*pyrR* (PyrR)	RMAR_RS00870	185941-185964
Rma_rrnA_f6	GGAGCATGTGGCTTAATTCGATGC	*rrsA* (16S rRNA)	RMAR_RS00885	188156-188179
Rma_rrnA_f7	GACGCGAAACCGGGTGAG	*rrlA* (23S rRNA)	RMAR_RS00900	189932-189949
Rma_rrnA_f8	GGTCCTAAGGTAGCGAAATTCC	*rrlA* (23S rRNA)	RMAR_RS00900	191195-191216
Rma_rrnA_r1	GAACGCACAAGGCGCGCGATCTATCC	*rrfA* (5S rRNA)	RMAR_RS00905	192478-192503
Rma_rrnA_r2	TCGTGGAGCAGCGTTTCGGCCATAGG	*purL* (PRFG synthase)	RMAR_RO00910	192532-192557
Rma_rrnA_r3	TCGACGATCCAGCCGTATTCTTCGTC	*purL* (PRFG synthase)	RMAR_RO00910	192601-192626
Rma_rrnA_r4	CTGTCGTTCCGCGAGTAGGCGGAG	*rrsA* (16S rRNA)	RMAR_RS00885	188770-188793
Rma_rrnA_r5	CCGAAGCTTCGGCGCCAGGCTTG	*rrlA* (23S rRNA)	RMAR_RS00900	190406-190428
Rma_rrnA_r6	GCACAACTGGTACACTAGAGG	*rrlA* (23S rRNA)	RMAR_RS00900	191954-191974
Rma_rrnA_r7	TTGCACTCTACGCACGATTACC	*rrlA* (23S rRNA)	RMAR_RS00900	191581-191602
Rma_rrnA_r8	CCTGATATCTACGCATTTCACC	*rrsA* (16S rRNA)	RMAR_RS00885	187903-187924
Rma_rplD_f1	CTGTAAACGGCATTGTCGAATTG	*rplD* (r-protein uL4)	RMAR_RS04205	965925-965947
Rma_rplD_r1	CGTCCATCTGCTGCGAGAGC	*rplD* (r-protein uL4)	RMAR_RS04205	966705-966724
Rma_rplV_f1	GGTCTATATCACGGAGAACATGGTC	*rplV* (r-protein uL22)	RMAR_RS04225	968074-968098
Rma_rplV_r1	CGCCCAGGCGAAATCCTATCGG	*rplV* (r-protein uL22)	RMAR_RS04225	968617-968638
Rma_rplK_f1	ACAGAAGCATTCGAGAGGCGC	*rplK* (r-protein uL11)	RMAR_RS05505	1275101-1275121
Rma_rplK_r1	TCTCAAGCGCCTGGCGATAGCG	*rplK* (r-protein uL11)	RMAR_RS05505	1275704-1275725
Rma_rpoB_f1	CTCTATCCAATTTGCAGGCG	*rpoB* (RNAP β-subunit)	RMAR_RS05525	1277636-1277655
Rma_rpoB_f2	CGCATGGCCCGTACCATC	*rpoB* (RNAP β-subunit)	RMAR_RS05525	1279042-1279059
Rma_rpoB_r1	TCTTCCCTTGCGGCATAGCG	*rpoB* (RNAP β-subunit)	RMAR_RS05525	1281600-1281619
Rma_rpoB_r2	CTTGACGAACTCGTTGAGG	*rpoB* (RNAP β-subunit)	RMAR_RS05525	1279521-1279539

Table S2. Antibiotic zones of inhibition. Discs were 6 mm in diameter.

**Antibiotic Class (Target)**	**Antibiotic**[1]	**Zone (mm)**
14-atom macrolide (ribosome, 50S)	erythromycin	41
	oleandomycin	26
	roxithromycin	46
	clarithromycin	53
15-atom macrolide (ribosome, 50S)	azithromycin	31
16-atom macrolide (ribosome, 50S)	spiramycin	35
	chalcomycin	18
	tylosin	59
	carbomycin	53
lincosamide (ribosome, 50S)	lincomycin	64
	clindamycin	71
streptogramin B (ribosome, 50S)	pristinamycin	51
pleuromutilin (ribosome, 50S)	tiamulin	44
amphenicol (ribosome, 50S)	chloramphenicol	19
thiopeptide (ribosome, 50S)	thiostrepton	43
elfamycin (EF-Tu)	kirromycin	38
fusidane (EF-G)	fusidic acid	36
rifamycin (RNA polymerase)	rifampicin	43
fluoroquinolone (DNA gyrase)	ofloxacin	41
	ciprofloxacin	46

[1] Antibiotics not listed produced no zones of inhibition.

References

17. Nolan, M.; Tindall, B.J.; Pomrenke, H.; Lapidus, A.; Copeland, A.; Glavina Del Rio T.; Lucas, S.; Chen, F.; Tice, H.; Cheng, J.F.; Saunders, E.; Han, C.; Bruce, D.; Goodwin, L.; Chain, P.; Pitluck, S.; Ovchinikova, G.; Pati, A.; Ivanova, N.; Mavromatis, K.; Chen, A.; Palaniappan, K.; Land, M.; Hauser, L.; Chang, Y.J.; Jeffries, C.D.; Brettin, T.; Göker, M.; Bristow, J.; Eisen, J.A.; Markowitz, V.; Hugenholtz, P.; Kyrpides, N.C.; Klenk, H.P.; Detter, J.C. 2009. Complete genome sequence of *Rhodothermus marinus* type strain (R-10). *Stand. Genomic Sci.* **2009**, *1*, 283-290.

54. Madeira F.; Park, Y.M.; Lee, J.; Buso, N.; Gur, T.; Madhusoodanan, N.; Basutkar, P.; Tivey, A.R.N.; Potter, S.C.; Finn, R.D.; Lopez, R. The EMBL-EBI search and sequence analysis tools APIs in 2019. *Nucleic Acids Res*. **2019,** *47*, W636-W641.
